# Synergy of *Rhizophagus intraradices* and Mycorrhiza Helper Bacteria in Enhancing Carbendazim Degradation and Soybean Growth Under Hydroponic and Soil Systems

**DOI:** 10.3390/plants15121833

**Published:** 2026-06-13

**Authors:** Tianzhao Guan, Yuying Lin, Yueqin Peng, Jingping Ge, Weiguang Jie, Wenxiang Ping

**Affiliations:** 1 Engineering Research Center of Agricultural Microbiology Technology, Ministry of Education & Heilongjiang Provincial Key Laboratory of Plant Genetic Engineering and Biological Fermentation Engineering for Cold Region & Key Laboratory of Microbiology, College of Heilongjiang Province & School of Life Sciences, Heilongjiang University, Harbin 150080, China; 17706883204@163.com (T.G.); pengyueqin1212@163.com (Y.P.); gejingping@126.com (J.G.); 2 School of Food Engineering, Heilongjiang East University, Harbin 150066, China; linyuying2023@126.com

**Keywords:** arbuscular mycorrhizal fungi, total number of bacterial colonies, soybean biomass, carbendazim residues, correlation analysis

## Abstract

Soybean is a critical economic, oil and industrial raw material crop, yet its production is often hindered by pathogen infection and pesticide residues. This study explored the synergistic effects of *Rhizophagus intraradices* and mycorrhizal helper bacteria (MHB) on AMF colonization, AMF spore density, total number of bacterial colonies, soybean growth, root rot disease index, and carbendazim residues. Hydroponic and pot experiments were conducted using a completely randomized design (CRD) with five biological replicates per treatment; after 30 days of growth, three replicates were randomly selected for all measurements. Results showed that inoculation with microbial agents, particularly co-inoculation, increased soybean biomass, reduced disease index, and decreased carbendazim residues. In the hydroponic experiment, co-inoculation increased plant height, aboveground fresh weight, and underground dry weight by 64.28%, 78.13%, and 109.09%, respectively, and decreased carbendazim residues by 71.84% relative to the carbendazim-alone group. In the pot experiment, co-inoculation reduced carbendazim residues by 81.25% and root rot disease index by 45.56% compared with the carbendazim-alone group. Correlation analysis showed a strong positive correlation (*p* < 0.001) between carbendazim degradation in hydroponic and pot systems, indicating stable degradation function across environments. Co-inoculation of *R. intraradices* and MHB synergistically promotes soybean growth, suppresses root rot, and reduces carbendazim residues, providing a theoretical basis for developing functional microbial inoculants for safe and green soybean production.

## 1. Introduction

Soybean (*Glycine max* L.) is a major economic crop worldwide and has high nutritional value. It is abundant in nutrients such as protein, fat and vitamins [1]. Over recent years, the acreage and production of soybeans in China have steadily increased. However, soybean root rot causes lodging and yield loss, resulting in substantial economic losses [2]. Soybean root rot is a fungal disease spread through soil, with a wide range of infection and a long infection cycle. It is not limited to a specific stage of soybean growth and development, but occurs at all stages [3]. The pathogens causing soybean root rot vary with the planting area, and fungi are the main pathogens. Among them, there are four core pathogenic fungi: *Fusarium* [4], *Phytophthora* [5], *Rhizoctonia* [6], and *Pythium* [7]. These pathogens collectively contribute to the incidence and severity of root rot, necessitating effective control strategies. For the control of soybean root rot, several agricultural practices are adopted, such as seed selection and seed coating [8], crop rotation, and ridge tillage [9]. However, in cases of severe disease, chemical pesticides are usually used to control root rot, and carbendazim is widely adopted for this purpose. Carbendazim is a broad-spectrum benzimidazole fungicide widely used to control fungal diseases in various crops. It is also often used as a preservative for grains and other agricultural products post-harvest [10]. However, long-term or excessive application of carbendazim can result in carbendazim residues, which pose severe threats to human health and the ecological environment [11]. The degradation methods of carbendazim residues mainly include photolysis, hydrolysis, chemical degradation, and biological degradation [12]. Among them, biological degradation is considered a promising, efficient, low-energy, and environmentally friendly approach for the sustainable management of carbendazim residues.

Arbuscular mycorrhizal fungi (AMF) are a widely distributed group of soil microorganisms in agricultural ecosystems. AMF establish obligate symbioses with more than 80% of land plants, performing multiple ecological functions within agricultural ecosystems [13,14]. *Rhizophagus intraradices* is one of the most extensively studied AMF belonging to Glomeromycota. It possesses unique symbiotic functions and significant ecological value [15]. *R. intraradices* exhibits rapid root colonization, developing arbuscules, hyphae, and spores within the cortex to ensure efficient nutrient exchange and propagation [16]. After establishing mutualistic symbiosis with plant roots, *R. intraradices* enhances crop nutrient acquisition from soil [17], improves crop disease resistance through induced systemic defense responses [18], and increases host plant tolerance to abiotic stress [19]. Additionally, *R. intraradices* modulates auxin, gibberellin, and abscisic acid to enhance root architecture and osmotic adjustment under salinity and heavy metal stress [20], and optimizes the rhizosphere soil microenvironment via hyphal networks and glomalin secretion, thereby promoting crop biomass accumulation. AMF commonly associate with mycorrhizal helper bacteria (MHB), such as *Pseudomonas* and *Bacillus*, which promote AMF growth, spore germination, and hyphal branching, thereby enhancing plant growth, nutrient uptake, and stress tolerance [21].

Mycorrhizal helper bacteria (MHB) are a group of functional bacteria widely distributed in the rhizosphere environment [22]. Their core functions include promoting the germination of AMF spores [23], stimulating hyphal growth and extension to establish symbiotic relationships with plant roots, increasing plant root branching, promoting the contact and colonization of AMF with plant roots [24], and alleviating the adverse effects of harsh environmental conditions on mycorrhizal fungal mycelium. *B. simplex* has strong sporulation capacity and efficient rhizosphere colonization, and its secreted hormones and cell wall-softening enzymes promote AMF penetration [25]; *F. ginsengisoli* possesses strong root affinity and improves rhizosphere nutrition by decomposing organic matter and synthesizing siderophores [26]. *B. megaterium* exhibits excellent phosphorus-solubilizing capacity and IAA synthesis ability [27], thereby promoting plant growth [28]. Meanwhile, *B. megaterium* strain HLJ7 can degrade pesticide [29]. Therefore, the co-inoculation of these three MHB strains with *R. intraradices* is expected to generate synergistic effects, providing microbial technological support for the green transformation of agriculture.

However, relevant research on the co-inoculation of *R. intraradices* and MHB regarding soybean growth, soybean root rot, and carbendazim residues remains limited. The present study aims to comprehensively investigate the synergistic effects of the co-inoculation of *R. intraradices* and MHB on (a) promoting soybean growth, (b) suppressing root rot, and (c) degrading carbendazim residues. To achieve these aims, hydroponic and pot experiments were established to evaluate the AMF colonization rate, AMF spore density, the total number of bacterial colonies, plant biomass, nodule number, the disease index of soybean root rot, and carbendazim residues, thereby providing a theoretical basis for the development of plant growth-promoting microbial inoculants and microbial remediation of pesticide-contaminated soil.

## 2. Results

### 2.1. Hydroponic Experiment: AMF Colonization, Soybean Growth, and Carbendazim Residues

#### 2.1.1. AMF Colonization and Total Number of Bacterial Colonies

**Figure 1:**
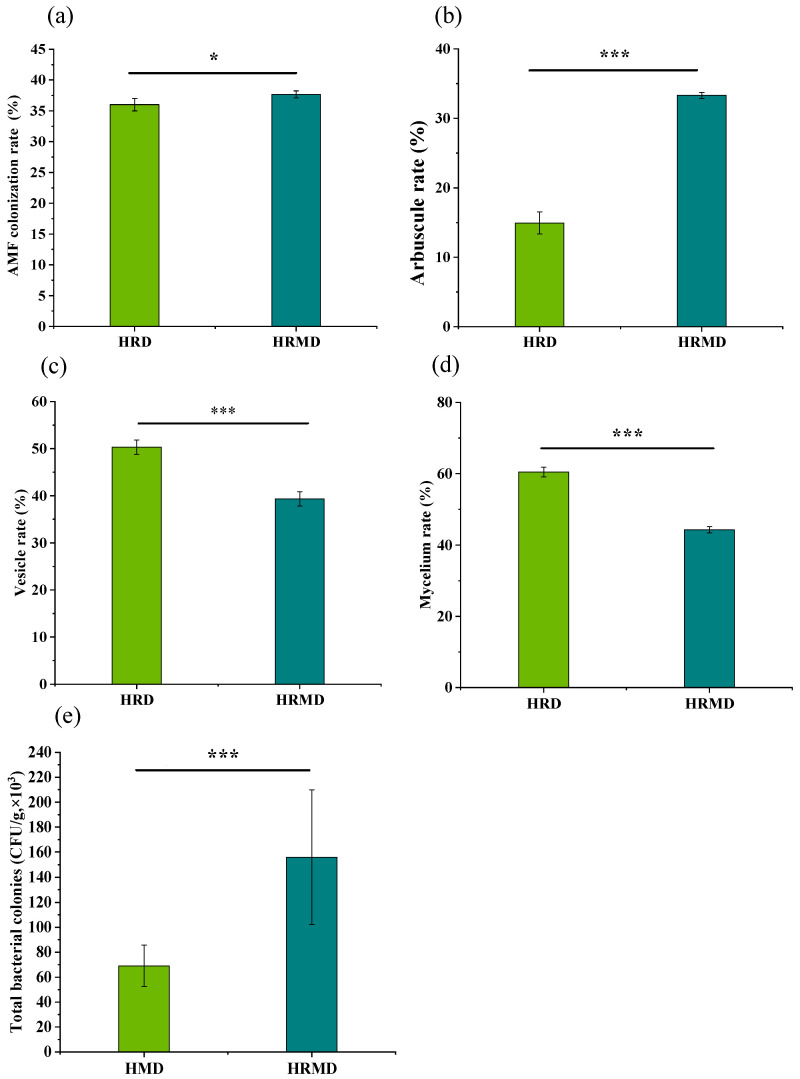
Effects of different treatment groups on AMF colonization in soybean roots and total number of bacterial colonies in the rhizosphere microenvironment in the hydroponic experiment. (**a**) AMF colonization rate; (**b**) arbuscule rate; (**c**) vesicle rate; (**d**) mycelium rate; (**e**) total number of bacterial colonies. Hydroponic experiment (H): HRD (carbendazim spraying + *R. intraradices* inoculation); HMD (carbendazim spraying + MHB inoculation); HRMD (carbendazim spraying + *R. intraradices* + MHB co-inoculation). Error bars indicate standard deviation (*n* = 3). * *p* < 0.05 indicates significant difference, ** *p* < 0.01 indicates relatively significant difference, *** *p* < 0.001 indicates extremely significant difference.

As shown in Figure 1a–d, the AMF colonization rate and arbuscule rate in the HRMD group were 5.60% and 122.89% higher than those in the HRD group, respectively. This indicates that co-inoculation of MHB and *R. intraradices* significantly improved the colonization efficiency and symbiotic function of AMF in soybean roots. In contrast, the mycelium rate and vesicle rate in the HRD group were 36.65% and 27.97% higher than those in the HRMD group, respectively. These results suggest that MHB have a regulatory effect on the formation of AMF structures, which is a functional manifestation of AMF adapting to different symbiotic environments. It can be concluded that MHB significantly promote the colonization of soybean roots by *R. intraradices* (*p* < 0.05), form a symbiotic relationship, and thus promote soybean growth.

As shown in Figure 1e, the total number of bacterial colonies in the HMD group reached 0.69 × 10^3^ CFU/mL, indicating that MHB can effectively colonize soybean roots. The total number of bacterial colonies in the HRMD group was significantly higher than that in the HMD group (*p* < 0.05), reaching 1.56 × 10^3^ CFU/mL. These results demonstrate that co-inoculation of MHB and *R. intraradices* can enrich the rhizosphere microorganisms of soybeans, improve the rhizosphere microenvironment, and promote soybean growth under the same culture conditions.

#### 2.1.2. Soybean Biomass

**Figure 2:**
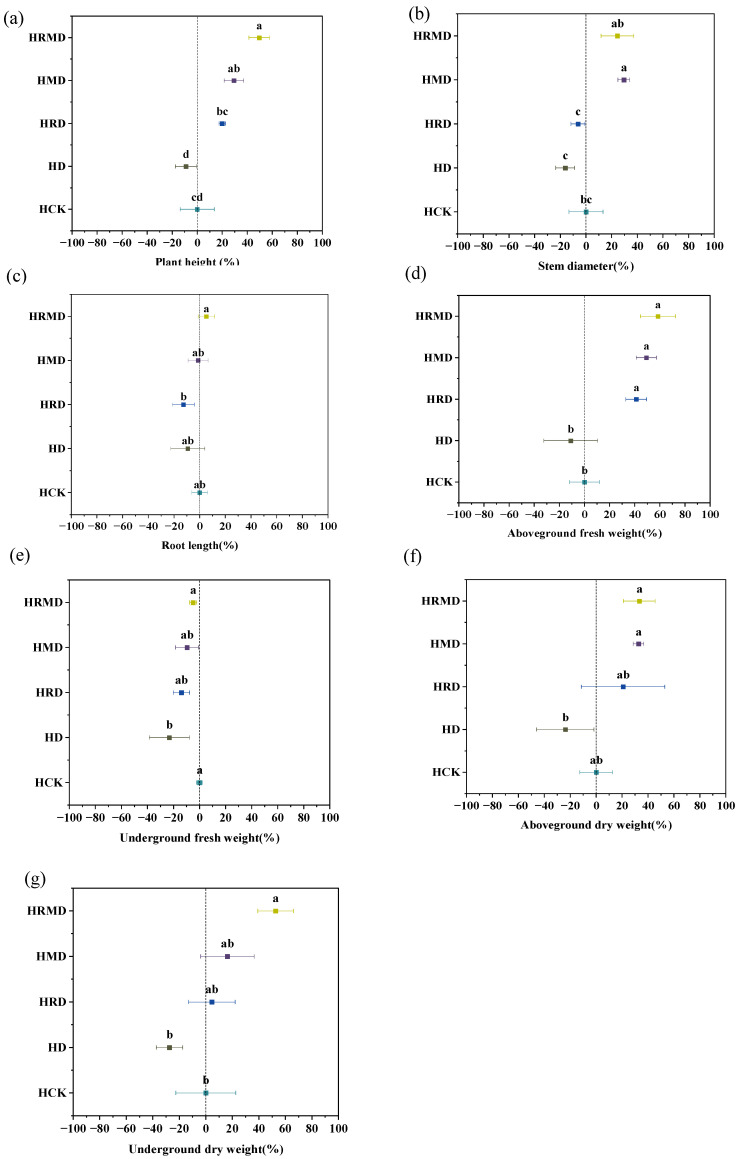
Effects of different treatment groups on soybean biomass in the hydroponic experiment. (**a**) Plant height; (**b**) Stem diameter; (**c**) Root length; (**d**) Aboveground fresh weight; (**e**) Underground fresh weight; (**f**) Aboveground dry weight; (**g**) Underground dry weight. Hydroponic experiment (H): HCK (blank control); HD (carbendazim spraying); HRD (carbendazim spraying + *R. intraradices* inoculation); HMD (carbendazim spraying + MHB inoculation); HRMD (carbendazim spraying + *R. intraradices* + MHB co-inoculation). The values in (**a**–**g**) were all calculated as follows: (treatment − CK)/CK × 100%. Error bars represent the 95% confidence interval (CI). A response was considered statistically significant when the 95% CI did not overlap with zero. Error bars indicate standard deviation (*n* = 3). Different letters indicate significant differences at *p* < 0.05 level.

Soybean plant height, stem diameter, root length, fresh weight, and dry weight were all improved in the single inoculation groups of *R. intraradices* or MHB, but the promotional effects were significantly lower than those in the co-inoculation group (*p* < 0.05) (Figure 2). Compared with the HCK group, the plant height, stem diameter, root length, aboveground fresh weight, underground fresh weight, aboveground dry weight and underground dry weight of soybean in the HD group all decreased by 8.94%, 16.03%, 9.35%, 10.96%, 23.13%, 23.93% and 26.67%, respectively. In addition, inoculation with *R. intraradices* or MHB alleviated the inhibitory effect of carbendazim, and the co-inoculation of *R. intraradices* and MHB achieved the best mitigating effect. Compared with the HD group, the HRMD group significantly increased the plant height, stem diameter, root length, aboveground fresh weight, underground fresh weight, aboveground dry weight and underground dry weight of soybean by 64.28%, 48.47%, 16.07%, 78.13%, 23.59%, 75.28% and 109.09%, respectively (*p* < 0.05).

#### 2.1.3. Carbendazim Residues

The effects of different treatment groups on carbendazim residues in the hydroponic experiment are shown in Table 1. The HD group had the highest carbendazim residues. Compared with the HD group, the carbendazim residues in the HRD, HMD and HRMD groups decreased by 67.14%, 64.15% and 71.84%, respectively. In comparison with the HRD and HMD groups, the carbendazim residues in the HRMD group were reduced by 14.31% and 21.47%, respectively. These results indicated that both *R. intraradices* and MHB were capable of degrading carbendazim under hydroponic conditions. MHB exhibited stronger environmental adaptability and higher degradation capacity than single inoculation with *R. intraradices*. In addition, the co-inoculation of *R. intraradices* and MHB exhibited the highest carbendazim degradation ability, while single inoculation of *R. intraradices* had the lowest degradation ability.

### 2.2. Pot Experiment: AMF Colonization, Soybean Growth, and Carbendazim Residues

#### 2.2.1. AMF Colonization, Spore Density and Total Number of Bacterial Colonies

**Figure 3:**
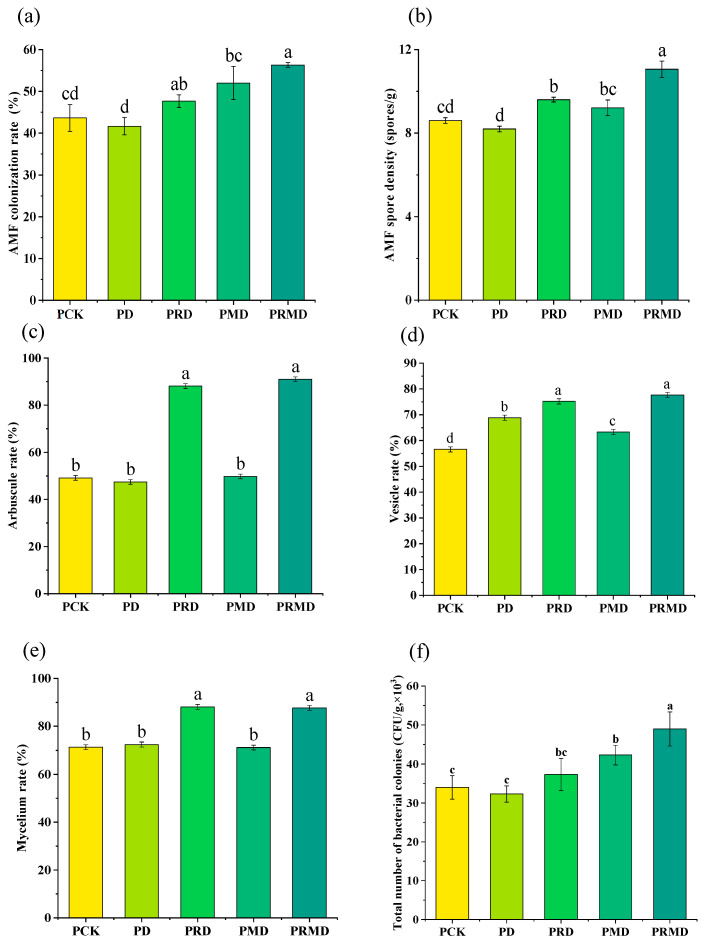
Effects of different treatment groups on AMF colonization in soybean roots and total number of bacterial colonies in rhizosphere soil in the pot experiment. (**a**) AMF colonization rate; (**b**) AMF spore density; (**c**) arbuscule rate; (**d**) vesicle rate; (**e**) mycelium rate; (**f**) total number of bacterial colonies. Pot experiment (P): PCK (blank control); PD (carbendazim spraying); PRD (carbendazim spraying + *R. intraradices* inoculation); PMD (carbendazim spraying + MHB inoculation); PRMD (carbendazim spraying + *R. intraradices* + MHB co-inoculation). Error bars indicate standard deviation (*n* = 3). Different letters indicate significant differences at *p* < 0.05 level.

As can be seen from Figure 3a, the AMF colonization rate was the highest in the PRMD group. Compared with the PCK group, the AMF colonization rate in the soybean roots in the PD group decreased by 4.58%, indicating that the carbendazim spraying treatment caused toxicity to the rhizosphere microorganisms of soybean roots, leading to a decrease in the AMF colonization rate. In comparison with the PD group, the AMF colonization rate in the PRD, PMD and PRMD groups was significantly increased by 14.40%, 24.80% and 35.20% (*p* < 0.05) (Figure 3a). The results suggested that inoculation with *R. intraradices* or MHB increased the AMF colonization rate of soybean roots, and the synergistic effect of the two strains yielded a more pronounced promotion. Thus, the addition of MHB significantly promoted the colonization of *R. intraradices* in soybean roots (*p* < 0.05), forming a symbiotic relationship and facilitating soybean growth.

AMF spore density directly reflects the reproductive capacity of *R. intraradices* in soil. As illustrated in Figure 3b, co-inoculation of *R. intraradices* and MHB significantly increased the AMF spore density (*p* < 0.05). The spore density in the PRMD group was 28.59% and 34.92% higher than that in the PCK group and PD group, respectively. These results demonstrated that the synergistic effect of *R. intraradices* and MHB promoted AMF spore germination and increased spore density.

As shown in Figure 3c–e, the AMF structural colonization characteristics of soybean roots exhibited significant differences among treatments. The arbuscule rate was significantly higher in the PRD and PRMD groups than in PCK (*p* < 0.05), indicating that the combination of AMF inoculation with MHB significantly promoted arbuscule formation. For the vesicle rate (Figure 3c), the PCK group showed the lowest level, while both PRD and PRMD treatment groups significantly increased vesicle formation (*p* < 0.05). The PMD group was significantly lower than the PRD and PRMD groups. Similarly, the mycelium rate (Figure 3d) was significantly enhanced in the PRD and PRMD groups compared with PCK, PD and PMD (*p* < 0.05), demonstrating that the co-inoculation of *R. intraradices* and MHB group effectively improved intraradical mycelium development in soybean roots.

Figure 3f showed the total number of bacterial colonies in the rhizosphere soil of soybean in the pot experiment under different treatment groups. The results indicated that different treatment groups had different effects on the total number of bacterial colonies in soybean rhizosphere soil. The total number of bacterial colonies in the PRMD group was significantly higher than that in the PCK and PD groups, increasing by 44.12% and 51.55% (*p* < 0.05), respectively. Meanwhile, the total number of bacterial colonies in the PRD and PMD groups was lower than that in the PRMD group, indicating that single strain has a weak ability to recruit rhizosphere soil bacteria. These results showed that the synergistic effect of *R. intraradices* and MHB increases the total number of bacterial colonies in the soybean rhizosphere soil.

#### 2.2.2. Soybean Biomass

**Figure 4:**
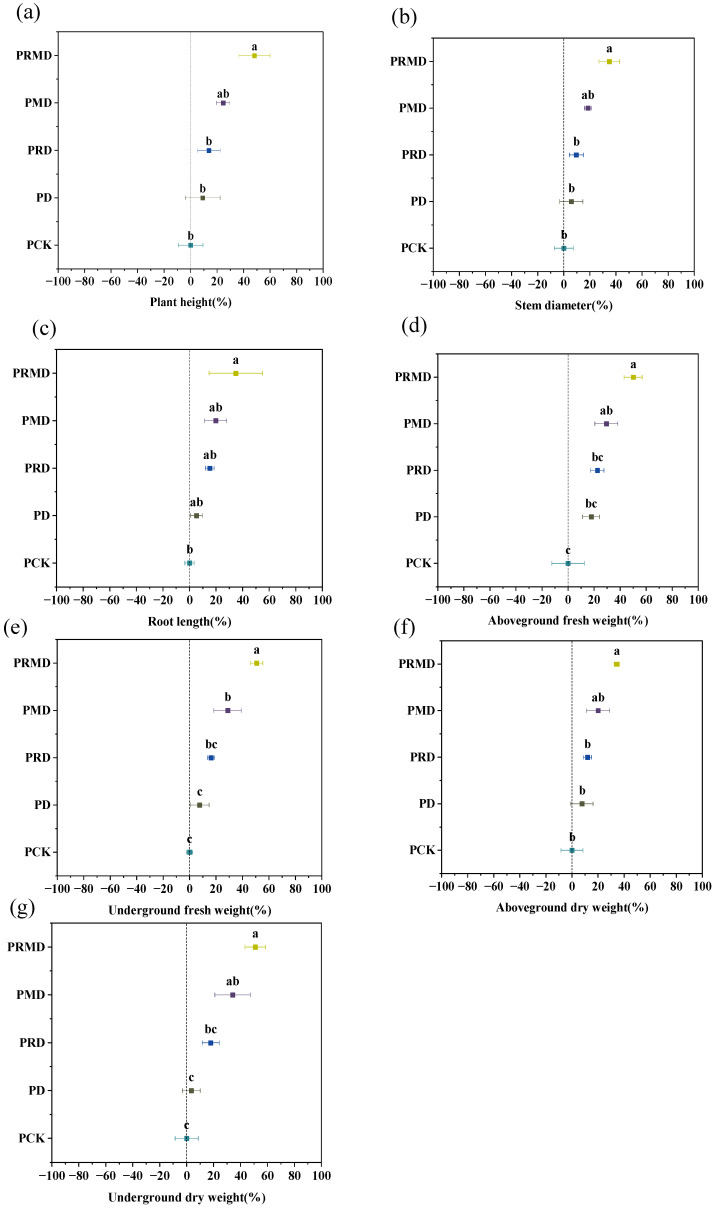
Effects of different treatment groups on soybean biomass in the pot experiment. (**a**) Plant height; (**b**) Stem diameter; (**c**) Root length; (**d**) Aboveground fresh weight; (**e**) Underground fresh weight; (**f**) Aboveground dry weight; (**g**) Underground dry weight. Pot experiment (P): PCK (blank control); PD (carbendazim spraying); PRD (carbendazim spraying + *R. intraradices* inoculation); PMD (carbendazim spraying + MHB inoculation); PRMD (carbendazim spraying + *R. intraradices* + MHB co-inoculation). The values in (**a**–**g**) were all calculated as follows: (treatment − CK)/CK × 100%. Error bars represent the 95% confidence interval (CI). A response was considered statistically significant when the 95% CI did not overlap with zero. Error bars indicate standard deviation (*n* = 3). Different letters indicate significant differences at *p* < 0.05 level.

As shown in Figure 4, the *R. intraradices* or MHB treatment groups significantly increased the plant height, stem diameter, root length, fresh weight and dry weight of soybean, but the effect was lower than that of the co-inoculation group (*p* < 0.05). Compared with the PCK group, the plant biomass in the PD group decreased significantly (*p* < 0.05), indicating that carbendazim has an obvious inhibitory effect on plant growth. In comparison with the PD group, the plant height, stem diameter, root length, aboveground fresh weight, underground fresh weight, aboveground dry weight and underground dry weight of soybean in the PRD group increased by 4.32%, 3.64%, 9.34%, 4.05%, 8.02%, 3.87%, and 13.33%, respectively. All the above biomass indices in the PMD group were higher than those in the PD group, increasing by 14.17%, 12.05%, 13.73%, 9.91%, 19.97%, 11.43% and 28.33%, respectively. These results suggested that inoculation with *R. intraradices* or MHB can increase soybean biomass, and co-inoculation of *R. intraradices* and MHB has the most significant promoting effect on soybean growth. The plant height, stem diameter, root length, aboveground fresh weight, underground fresh weight, aboveground dry weight and underground dry weight of soybean in the PRMD group were 35.78%, 27.50%, 28.07%, 27.44%, 40.08%, 24.74%, and 45.00% higher than those in the PD group, respectively (*p* < 0.05). These results indicated that co-inoculation of *R. intraradices* and MHB has the greatest advantage in promoting soybean biomass accumulation.

#### 2.2.3. The Number of Soybean Root Nodules and the Disease Index of Soybean Root Rot

**Figure 5:**
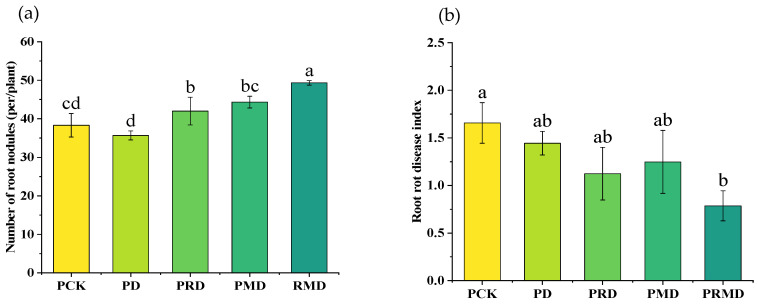
Effects of different treatment groups on the number of soybean root nodules (**a**) and the disease index of soybean root rot (**b**) in the pot experiment. Pot experiment (P): PCK (blank control); PD (carbendazim spraying); PRD (carbendazim spraying + *R. intraradices* inoculation); PMD (carbendazim spraying + MHB inoculation); PRMD (carbendazim spraying + *R. intraradices* + MHB co-inoculation). Error bars indicate standard deviation (*n* = 3). Different letters indicate significant differences at *p* < 0.05 level.

Figure 5a showed the effects of different treatment groups on the number of soybean root nodules. The results indicated that inoculation with *R. intraradices* or MHB could increase the number of soybean root nodules, and co-inoculation of *R. intraradices* and MHB had the most significant increase (*p* < 0.05). Compared with the PCK group and PD group, the number of soybean root nodules in the PRD and PMD groups was increased, with the PRD group increasing by 9.57% and 17.76%, and the PMD group increasing by 15.65% and 24.30%, respectively. The number of soybean root nodules in the PRMD group was 22.30% and 27.70% higher than that in the PCK group and PD group, respectively. These results demonstrated that both *R. intraradices* and MHB can recruit beneficial bacteria in the soybean rhizosphere, effectively promote the growth of rhizobia, and increase the number of soybean root nodules.

Figure 5b showed the effects of different treatment groups on the disease index of soybean root rot. The results showed that different treatment groups had different effects on the disease index of soybean root rot. Inoculation with *R. intraradices* or MHB significantly reduced the disease index of soybean root rot, and co-inoculation of *R. intraradices* and MHB had a better reduction effect (*p* < 0.05). Compared with the PCK group and PD group, the disease index of soybean root rot in the PRMD group was significantly decreased (*p* < 0.05) by 52.57% and 45.56%, respectively, indicating that *R. intraradices* and MHB can inhibit the growth and enrichment of soybean root rot fungi and reduce the disease index of root rot.

#### 2.2.4. Carbendazim Residues

The effects of different treatment groups on carbendazim residues in the pot experiment are shown in Table 2. The PD group had the highest carbendazim residues. Compared with the PD group, the carbendazim residues in the PRD, PMD and PRMD groups decreased by 80.38%, 60.77% and 81.25%, respectively, with the PRMD group having the lowest carbendazim residues. In comparison with the PRD and PMD groups, the carbendazim residues in the PRMD group were reduced by 4.40% and 52.20%, respectively. These results indicated that inoculation with *R. intraradices* or MHB can degrade carbendazim under the same culture conditions, with co-inoculation of *R. intraradices* and MHB having the best degradation ability. The degradation ability of the PRD group was higher than that of the PMD group, suggesting that *R. intraradices* has a stronger degradation ability than MHB in soil.

### 2.3. Correlation Analysis Between Hydroponic and Pot Experiments

**Figure 6:**
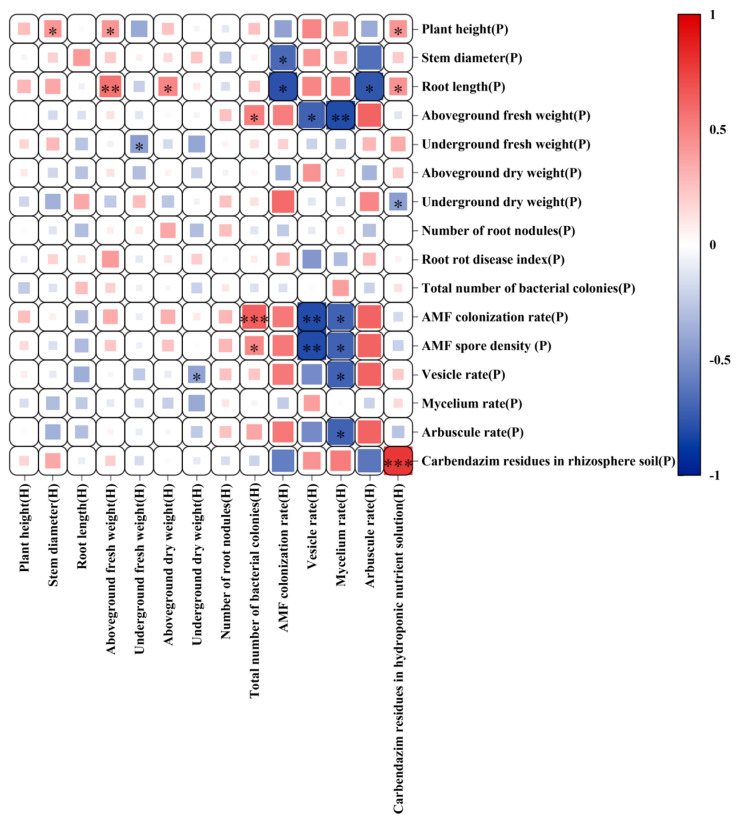
Spearman correlation analysis of AMF colonization rate, soybean biomass, the disease index of soybean root rot. and carbendazim residues in both hydroponic and pot experiments. The sample size (*n* = 15, from 5 treatments × 3 replicates). In the heatmap, red and blue represent positive and negative correlations respectively. (H) represents hydroponic experiment, and (P) represents pot experiment. * *p* < 0.05 indicates significant difference, ** *p* < 0.01 indicates relatively significant difference, *** *p* < 0.001 indicates extremely significant difference. Note: indicates *p* < 0.05. Small r values represent weak linear correlations; significance does not imply strong correlation strength.

Based on the data from all treatment groups in both hydroponic (HCK, HD, HRD, HMD, HRMD) and pot (PCK, PD, PRD, PMD, PRMD), Spearman correlation analysis was conducted on the AMF colonization rate, soybean biomass, the disease index of soybean root rot, and carbendazim residues in both the hydroponic and pot experiments. The results were shown in Figure 6. The carbendazim residues in the hydroponic nutrient solutions were highly significantly positively correlated with those in the pot culture (*p* < 0.001). This suggests that despite substantial differences in environmental complexity between the two systems, the microbial inoculant maintains highly consistent carbendazim degradation performance across different cultivation conditions, indicating that its degradation function is weakly influenced by environmental complexity. The aboveground fresh weight, AMF colonization rate, and AMF spore density in pot experiments were significantly negatively correlated with the vesicle rate and mycelium rate of hydroponic experiment (*p* < 0.05). The symbiotic structures of AMF and the soybean growth responses in the two culture systems are regulated by different mechanisms, which may be related to the lack of soil matrix in the hydroponic environment. AMF colonization rate in the pot experiment and the total number of bacterial colonies in the hydroponic experiment showed a highly significant positive correlation (*p* < 0.001). This reveals that the positive association between AMF colonization and bacterial abundance maintains a stable synergistic relationship across different cultivation environments.

These results indicated that the carbendazim degradation function of the microbial agent remained stable under different environmental complexities with favorable application reliability. The relationship between AMF colonization characteristics and soybean biomass was opposite in the hydroponic and the pot experiments, suggesting that the absolute colonization rates from the two experiments should not be directly compared. The AMF colonization rate in pot experiment can serve as a potential indicator of bacterial abundance in the hydroponic experiment, reflecting the coupled relationship of microorganisms across environments.

## 3. Discussion

In this study, hydroponic and pot experiments were conducted to systematically investigate the synergistic effects of *R. intraradices* and MHB on AMF colonization, soybean growth, disease control and pesticide degradation from a controlled environment to natural conditions. The hydroponic experiment can effectively eliminate the interference of indigenous microorganisms, organic matter and complex substrates in soil, which facilitates the precise control of inoculation amount of single strain and is conducive to the detection of carbendazim residues. The pot experiment uses natural soil as the substrate, which is closer to the actual field planting environment and can truly reflect the interaction process of rhizosphere soil microorganisms. The two experiments mutually verified the experimental results. This study highlighted the advantages of the synergistic effect of fungi and bacteria in agricultural production, laying a theoretical and practical foundation for subsequent field trials. The ultimate goal is to develop a green microbial inoculant suitable for soybean-producing areas in Heilongjiang Province, thereby addressing the issues of soil-borne diseases and pesticide residues.

Studies have demonstrated that MHB is crucial for the growth, colonization and functional differentiation of *R. intraradices*: it can provide carbon sources and activate nutrients, reduce the resource consumption of AMF hyphal extension and vesicle accumulation, promote the differentiation of AMF toward strengthening symbiotic interaction, and improve the efficiency of nutrient exchange. The hydroponic experiment in this study found that the colonization rate and arbuscule rate in the HRMD group were 5.60% and 122.89% higher than those in the HRD group, while the hyphal rate and vesicle rate were 36.65% and 27.97% lower, respectively, indicating that MHB plays an important role in the colonization and growth of *R. intraradices*. Meanwhile, MHB creates a suitable colonization microenvironment for *R. intraradices* by recruiting rhizosphere microbial communities, thus improving its spore germination rate and colonization stability. In the pot experiment, the AMF spore density in the PRMD group was 34.92% higher than that in the PD group, which is consistent with the results of Bourles et al. [30]. In addition, *R. intraradices* can improve the enrichment ability of MHB. The continuous extension of *R. intraradices* hyphae can increase the contact efficiency of MHB with roots and AMF [31]. In the hydroponic experiment, the total number of bacteria in the HRMD group (1.56 × 10^3^ CFU/mL) was significantly higher than that in the HMD group (0.69 × 10^3^ CFU/mL), indicating that *R. intraradices* has an obvious promoting effect on the enrichment of MHB. Studies have shown that inoculation with *R. intraradices* can significantly increase soybean biomass, improve the rhizosphere microbial community, and reduce the disease index of root rot [32,33]. Both hydroponic and pot experiments in this study confirmed that single inoculation with *R. intraradices* or MHB had a certain effect on increasing soybean biomass and reducing the disease index of soybean root rot, but the effect was lower than that of co-inoculation with *R. intraradices* and MHB. In the pot experiment, compared with the PD group, the plant height, stem diameter, root length, aboveground fresh weight, underground fresh weight, aboveground dry weight, underground dry weight and number of soybean root nodules in the PRMD group increased by 35.78%, 27.50%, 28.07%, 27.44%, 40.08%, 24.74%, 45.00% and 27.70%, respectively (*p* < 0.05), and the disease index of soybean root rot decreased by 45.56%. These results indicated that the synergistic regulation of the two strains on the root microecology and growth and development of soybean is extremely significant.

The synergistic effect of *R. intraradices* and MHB not only promotes plant growth but also has the function of degrading pesticide residues [34]. Wang et al. [35] reported that AMF can decompose soil pollutants through various pathways, such as absorbing pollutants, enhancing the activity of enzymes related to pollutant degradation, and promoting the activity of microorganisms that decompose pollutants in soil. For example, Huang et al. [36] found that AMF inoculation can enhance the dissipation of atrazine in the rhizosphere and bulk soil of maize. Chen et al. [37] found that AMF can degrade tebuconazole. In addition, MHB can also degrade pesticide residues through direct and indirect effects [38,39]. Zhao et al. [40] found that *Bacillus licheniformis* can degrade 3-phenoxybenzoic acid, a toxic intermediate of the pesticide beta-cypermethrin. The hydroponic experiment showed that the carbendazim residues in the HD group were significantly higher than those in the HRD, HMD and HRMD groups, and the HMD group had higher degradation ability and environmental adaptability than the HRD group. These results indicated that inoculation with *R. intraradices* or MHB has the potential to degrade pesticides, and the hydroponic environment is more conducive to the colonization and degradation function of MHB. In contrast, the respiration and growth of *R. intraradices* hyphae are disturbed, and their attachment is limited, leading to the restriction of pesticide degradation [41]. In the pot experiment, the PD group had the highest carbendazim residues and the PRMD group had the lowest, and the degradation ability of the PRD group was higher than that of the PMD group. This indicated that soil can provide an attachment carrier and nutrients for *R. intraradices*, promote the extension of its hyphae and the exertion of its degradation function, while the colonization and degradation efficiency of MHB are inhibited by factors such as the competition of indigenous microorganisms. However, both hydroponic and pot experiments confirmed that the co-inoculation of *R. intraradices* and MHB had the best pesticide degradation effect. *R. intraradices* and MHB have a synergistic and complementary effect. MHB provides a guarantee for the growth and colonization of *R. intraradices* by activating nutrients and optimizing the microenvironment, and *R. intraradices* expands the contact range with MHB through the continuous extension of hyphae. The cooperation and complementarity of the two strains improve the degradation efficiency of carbendazim, and this synergistic effect can stably exist in both hydroponic and pot environments. These results also provide a key basis for the subsequent development of green microbial inoculants, especially in the field soil environment. Their synergistic effect can not only promote soybean growth and prevent and control soybean soil-borne diseases but also efficiently degrade carbendazim residues. However, further in-depth mechanism studies are still needed to provide a more solid theoretical support and practical technology for pesticide biodegradation and the development of green agriculture.

Based on Spearman correlation analysis, this study systematically explored the correlations among AMF colonization rate, soybean biomass, the disease index of soybean root rot and carbendazim residues in both hydroponic and pot experiments. The results revealed that the capacity of AMF to synergistically promote carbendazim degradation exhibits favorable environmental stability, and different cultivation modes can differentially regulate the interactive relationships between soybean and symbiotic microorganisms. Among them, the carbendazim residues in hydroponic nutrient solutions were extremely significantly positively correlated with those in pot soil (*p* < 0.001), indicating that the degradation capacity of the tested microbial agent for carbendazim presented a high degree of consistency across different cultivation systems. Pot experiments feature a complex soil matrix, higher microbial diversity and stronger adsorption capacity, whereas hydroponic systems have simpler nutrient compositions and controllable environmental interference. Even so, the degradation performance of the microbial inoculant is barely affected by environmental complexity, demonstrating excellent functional stability and application potential. This finding confirms that the degradation and metabolic pathways of benzimidazole pesticides mediated by microbial agents are weakly disturbed by habitat conditions, enabling stable pollutant mineralization potential under differentiated cultivation modes [42]. It provides theoretical support for the diversified application of this compound microbial agent in soybean planting systems. Such functional stability has also been reported in studies on AMF-assisted degradation of other persistent organic pollutants [43,44]. AMF contribute to the degradation of polycyclic aromatic hydrocarbons (PAHs) [45]. Mycorrhizae can increase hydrogen peroxide content in plant roots and enhance the activities of oxidases in roots and rhizosphere soil, thereby promoting the degradation of polycyclic aromatic hydrocarbons. Furthermore, previous studies have confirmed that *Bacillus* species can degrade carbendazim via hydrolases and cytochrome P450 enzymes [46]. *Flavobacterium* species also possess the ability to metabolize various pesticides [47,48]. These findings suggest that *R. intraradices* and the MHB strains (*Bacillus simplex*, *Bacillus megaterium*, and *Flavobacterium ginsengisoli*) have pesticide-degrading potential.

Moreover, the aboveground fresh weight, AMF colonization and AMF spore density in pot experiments were significantly negatively correlated with vesicle and mycelium rates in the hydroponic group (*p* < 0.05). Soil experiments can create a stable microecological environment for AMF hyphal colonization, spore germination and arbuscule development, thereby synergistically improving soybean biomass. In comparison, hydroponic experiments are devoid of soil particles and organic substrates, which reshape the carbon allocation patterns and symbiotic structural differentiation characteristics of AMF. This stimulates the massive proliferation of vesicles and free mycelium, and weakens the growth-promoting effect of mycorrhizae on soybean [49]. These distinct correlation patterns clearly indicate that absolute AMF colonization parameters derived from hydroponic and pot experiments are not directly comparable. Consistent cultivation regimes and substrate conditions are therefore required for accurate functional evaluation of mycorrhizal fungi.

In the pot experiment, the AMF colonization rate exhibited an extremely significant positive correlation with the total number of bacterial colonies in the hydroponic experiments (*p* < 0.001), confirming an intrinsic relationship between AMF and plant growth-promoting rhizobacteria across diverse cultivation environments. AMF can reshape the rhizosphere microecological structure through the extension of hyphal networks, regulation of root exudates, and acidification of the rhizosphere microenvironment, thereby providing a favorable microhabitat for the proliferation and colonization of beneficial bacteria [50,51]. AMF secretions and hyphal surfaces can create the “mycorrhizosphere” effect to selectively stimulate bacterial proliferation. Such interactions have been well documented in soil environments [52] and remain stable even when AMF hyphae grow in hydroponic nutrient solutions. This strong correlation is unaffected by cultivation patterns (soil culture vs. hydroponic culture), revealing that the symbiotic interaction between AMF and rhizosphere bacteria is highly environmentally stable. This association mainly depends on soluble signaling molecules rather than soil particle adhesion. Therefore, AMF colonization level in pot systems can reliably reflect rhizosphere bacterial abundance in hydroponic systems, and can be used as an effective indirect indicator of microbial functional activity.

In conclusion, this study verified that the composite microbial inoculant maintains stable degradation capacity against carbendazim in hydroponic and pot experiments, showing promising potential for field application. However, several limitations should be acknowledged. First, we acknowledge that the 30-day period after soybean emergence, while critical for AMF symbiosis establishment and pesticide sensitivity [53,54], is considerably shorter than the time required to reach harvest. Therefore, in future investigations, we will monitor the entire growth cycle of soybean to provide a more comprehensive assessment. Second, under controlled conditions, the synergistic effects were consistently observed within 30 days after soybean emergence. The fact that these effects were reproducible across both hydroponic and pot systems strongly indicates their stability. Building on these results, future field studies are necessary to confirm whether these effects are similarly effective under real cropping conditions. Third, while carbendazim residues were quantified, the detection of its key derivative, 2-aminobenzimidazole (2-AB), was not performed. Consequently, future studies will include comprehensive metabolite detection methods to monitor both the parent compound and its derivatives.

## 4. Materials and Methods

### 4.1. Location and Soil Sampling

The experiment was carried out at the experimental station in Pingfang District, Harbin, Heilongjiang Province, China (126°65′ E, 45°57′ N). The region is characterized by a moderate temperate continental monsoon climate, with an annual mean temperature of 4.2 °C, annual precipitation of 530 mm, and a frost-free period of 135–145 days. The experimental soil was taken from the 0–20 cm layer of a local soybean experimental field, then the soil was sieved to remove large particles.

### 4.2. Materials

#### 4.2.1. Soybean Material and Seed Germination

The non-genetically modified soybean variety Heinong 48 (HN48), which is widely cultivated in Heilongjiang Province, was selected as the experimental material. This variety is a disease-sensitive and high-protein cultivar, with an average protein content of 45.23% and an average fat content of 19.50%. The soybean seeds were purchased from the Heilongjiang Academy of Agricultural Sciences. Plump, uniformly sized soybean seeds with intact seed coats were selected, sterilized with chlorine gas for 15 h [55], then inoculated onto 1.5% water agar medium, and cultured in the dark at 25 °C for 7 d.

#### 4.2.2. Carbendazim Material and Solution Preparation

Carbendazim, with the chemical name methyl 2-benzimidazolecarbamate, has the molecular formula C_9_H_9_N_3_O_2_ and a relative molecular mass of 191.2. As a systemic benzimidazole fungicide, it exhibits broad-spectrum antifungal activity against various plant pathogens. The carbendazim product used in this study was a 50% wettable powder purchased from Tianjin Kemiou Co., Ltd. (Tianjin, China). Before use, 3.6 g of carbendazim powder was fully dissolved in 900 mL of sterile water, resulting in an effective concentration of 0.002 g/mL.

#### 4.2.3. Preparation of AMF Inoculant

The arbuscular mycorrhizal fungus *Rhizophagus intraradices* was provided by the Key Laboratory of Microbiology, Heilongjiang University. *R. intraradices* was propagated using alfalfa (*Medicago sativa* L.) for approximately 5 months to obtain the inoculant. The propagation substrate consisted of soil, sand, and vermiculite at a volume ratio of 5:2:3 (*v*/*v*/*v*). The soil, sand, and vermiculite used in the propagation substrate were all autoclaved. The soil was the same as that used in the pot experiment. The sand (0.50–0.71 mm) and vermiculite (1–3 mm) have negligible effects on soybean growth. The specific preparation method was based on the protocol described by Jie et al. [56].

#### 4.2.4. Preparation of MHB Inoculant

The MHB inoculant comprised three bacterial strains, *Bacillus simplex*, *Bacillus megaterium*, and *Flavobacterium ginsengisoli*. These strains were provided by the Key Laboratory of Microbiology, Heilongjiang University. Each bacterial strain was individually inoculated into LB liquid medium and cultured at 28 °C for 24 h. After centrifugation at 8000 rpm for 10 min, the bacterial pellets were collected, resuspended in sterile water to prepare bacterial suspensions, and the effective concentration of each suspension was adjusted to 1 × 10^8^ CFU/mL. Finally, the three bacterial suspensions were mixed in equal volumes to obtain the MHB inoculant.

#### 4.2.5. Hydroponic Nutrient Solution

Modified Hoagland’s nutrient solution (Phygene Biotechnology, Cat. No. PH1782, Fuzhou, China) was used for the hydroponic culture of soybean seedlings. Containing both nitrate and ammonium nitrogen, this solution was well suited for soybean culture and rhizosphere microbial interaction assays. To prepare the working solution, 1.26 g of the base nutrient powder and 0.945 g of calcium salt were weighed and fully dissolved in 1000 mL of distilled water with heating. The finished solution contained 945 mg/L Ca (NO_3_)_2_·4H_2_O, 506 mg/L KNO_3_, 80 mg/L NH_4_NO_3_, 136 mg/L KH_2_PO_4_ and 493 mg/L MgSO_4_·7H_2_O, supplemented with standard levels of chelated iron and trace elements [57]. The pH of the nutrient solution was set to 5.8 ± 0.2, and the solution was refreshed every 7–10 days during cultivation to maintain stable seedling growth conditions.

#### 4.2.6. Soil Media and Its Characteristics

The tested soil was typical black soil, classified as Mollisols according to the United States Department of Agriculture (USDA) Soil Taxonomy. Its physicochemical properties were as follows: pH 7.12 ± 0.21, organic matter 26.72 ± 1.15 g⋅kg^−1^, total nitrogen 1.21 ± 0.13 g⋅kg^−1^, total phosphorus 0.87 ± 0.09 g⋅kg^−1^, total potassium 23.85 ± 0.73 g⋅kg^−1^. The contents of loam, clay and sand particles were 34.27 ± 0.28%, 24.25 ± 0.17% and 41.19 ± 0.57%, respectively.

### 4.3. Experimental Design and Procedures

#### 4.3.1. Hydroponic Bioassay and Crop Establishment

A total of five treatment groups were set up for the hydroponic experiment (H): blank control group (HCK); carbendazim spraying group (HD); carbendazim spraying combined with *R. intraradices* inoculation group (HRD); carbendazim spraying combined with MHB inoculation group (HMD); carbendazim spraying combined with co-inoculation of *R. intraradices* and MHB group (HRMD). The experiment was arranged in a completely randomized design (CRD). Each treatment had five biological replicates, with a total of 25 flasks, and 2 soybean seedlings were cultured in each flask. After 30 days of growth, three flasks were randomly selected from each treatment for all subsequent measurements. For each flask, the two seedlings were averaged to obtain a single replicate value. All data are expressed as mean ± standard deviation (*n* = 3).

Germinated soybean seedlings were transplanted into Hoagland solution [58]. For AMF treatment groups (HRD, HRMD), *R. intraradices* spores were isolated by the wet-sieve decantation and sucrose centrifugation method. The isolated spores were surface-sterilized in 3% sodium hypochlorite solution for 3 min and rinsed with sterile water. Subsequently, 30 spores were transferred into culture flasks containing soybean seedlings. MHB inoculation was performed as described previously by Jie et al. [35]. 5 mL of MHB inoculant was injected into the hydroponic nutrient solution with a sterile syringe after transplanting soybean seedlings; an equal volume of sterile water was injected into the other treatment groups as the control. Carbendazim was added to the nutrient solution for the carbendazim treatment groups (HD, HRD, HMD, HRMD), whereas the blank control group (HCK) was cultured in standard nutrient solution without carbendazim.

All plants were harvested on day 30 after seedling emergence, at which time they had reached the V5 growth stage, characterized by 1–2 obvious main-stem branches, fully unfolded trifoliate leaves, rapid stem growth and active root development, making the rhizosphere microenvironment sufficiently representative for analysis. At the time of sampling, five plants and their corresponding nutrient solutions were randomly collected from each treatment to determine the following indices in the hydroponic experiment: the AMF colonization rate and colonization structures in soybean roots, total number of bacterial colonies in the soybean nutrient solutions, soybean biomass, and the residual carbendazim content.

#### 4.3.2. Pot Experiment and Procedure

Five treatment groups were also established for the pot experiment (P): blank control group (PCK); carbendazim spraying group (PD); carbendazim spraying combined with *R. intraradices* inoculation group (PRD); carbendazim spraying combined with MHB inoculation group (PMD); carbendazim spraying combined with co-inoculation of *R. intraradices* and MHB group (PRMD). The experiment was arranged in a completely randomized design (CRD). Each treatment group consisted of five biological replicates, with a total of 25 pots. Six soybean seeds were sown in each pot, and seedlings were thinned to three uniform plants per pot after emergence. After 30 days of growth, three pots were randomly selected from each treatment for all subsequent measurements. For each selected pot, the three plants were averaged to obtain a single replicate value. All data are expressed as mean ± standard deviation (*n* = 3).

*R. intraradices* inoculation was performed as described previously by Jie et al. [59]. For AMF treatment groups (PRD, PRMD), 50 g of *R. intraradices* inoculant was uniformly mixed into pot soil before sowing, followed by covering with 4–5 cm of soil; six soybean seeds were sown per pot, covered with 2–3 cm of soil and irrigated appropriately. The other groups received the same weight of ordinary soil without *R. intraradices* inoculant. Carbendazim solution was added to the carbendazim treatment groups (PD, PRD, PMD, PRMD) at sowing. The root drenching method was adopted for the MHB inoculation groups (PMD, PRMD): the prepared bacterial suspension (1 × 10^8^ CFU/mL) was applied around the soybean roots at a volume of 5 mL per plant with a sterile syringe, then covered with 2–3 cm of soil cover and sprinkled with sterile water to maintain soil moisture. The blank control group was irrigated with the same volume of sterile water.

Referring to the method of Zhou et al. [60], sampling of the pot experiment was performed at 30 d after soybean emergence, with 3 pots and their rhizosphere soil randomly collected from each treatment. The following indices were measured in the pot experiment: AMF colonization rate in soybean roots, AMF spore density in soybean rhizosphere soil, total number of bacterial colonies in soybean rhizosphere soil, soybean biomass, number of soybean root nodules, the disease index of soybean root rot, and carbendazim residues.

### 4.4. Measurement of Response Variables

#### 4.4.1. AMF Colonization Rate and Colonization Structures

The alkali separation-acid fuchsin method was used to determine the AMF colonization rate and colonization structures according to Jie et al. [61].

#### 4.4.2. AMF Spore Density

AMF spores were extracted by wet sieve decanting and sucrose centrifugation and the spore density was determined by the direct counting method [62].

#### 4.4.3. Total Number of Bacterial Colonies

In the hydroponic experiment, the total number of bacterial colonies attached to soybean roots was determined. Soybean seedlings were removed from the hydroponic planting holes, and the roots were gently rinsed with sterile deionized water. The rinsed roots were placed in a flask containing 225 mL of sterile deionized water, followed by gentle shaking to detach the microorganisms tightly adhering to the root surfaces. The suspension in the Erlenmeyer flask was filtered through a sterile membrane filter, and the microorganisms on the membrane were collected for subsequent determination.

In the pot experiment, the total number of bacterial colonies in soybean rhizosphere soil was measured. A 25 g soil sample was collected from the pot treatment and transferred into a flask containing 225 mL of sterile deionized water, followed by shaking at 28 °C with a rotation speed of 180 r/min for 30 min. The soil homogenate was serially diluted to 10^−3^, 10^−4^ and 10^−5^ with sterile deionized water, and 0.2 mL of each dilution was evenly spread onto the surface of beef extract peptone agar medium (3 g beef extract, 10 g peptone, 5 g NaCl, 20 g agar, 1 L distilled water, pH 7.4–7.6). The plates were incubated at 28 °C for 48 h, and the total number of culturable bacterial colonies was quantified [63].

#### 4.4.4. Soybean Biomass

The plant height, stem diameter, root length, aboveground fresh weight, underground fresh weight, aboveground dry weight and underground dry weight of soybean plants were determined according to the method of Rotundo et al. [64].

#### 4.4.5. The Number of Soybean Root Nodules

Soybean roots were completely excavated by the traditional digging method, and the soil attached to the root surface was gently removed. The roots were slowly rinsed with clean water and drained thoroughly after washing. All soybean root nodules were detached from the roots of each plant, and the number of soybean root nodules was counted.

#### 4.4.6. The Disease Index of Soybean Root Rot

The occurrence of soybean root rot was recorded by observing the symptoms of soybean plants in the field. The disease was characterized by necrosis of the hypocotyl, roots, stems and cotyledons of soybean. The disease index of soybean root rot was determined following the method described previously by Cui et al. [65]. The scoring standard for soybean root rot disease index was as follows: 0, absence of lesions on basal stems and taproots; 1, scattered lesions; 2, coalesced lesions; 3, lesions covering 25% of the root length; 4, lesions covering 33% of the root length and girdling the stem base, but without root necrosis; 5, lesions covering over 50% of the root length.

The disease index was calculated using the formula [66]:

Disease Index = 100 × [∑ (Number of diseased plants × Disease score)]/(Total plants × Maximum score). Where score values correspond to 0–5 as defined above.

#### 4.4.7. Carbendazim Residues

Preparation of carbendazim standard solution: A precise 50 mg of carbendazim standard (analytical grade, Tianjin Kermel, accurate to 0.0001 g) was weighed using a one-ten-thousandth analytical balance, and transferred into a 100 mL volumetric flask. 5 mL of methanol (HPLC grade, Tianjin Kermel Chemical Reagent Co., Ltd., Tianjin, China) and 1 mL of glacial acetic acid (analytical grade, Tianjin Guangfu Technology Development Co., Ltd., Tianjin, China) were added to dissolve the standard thoroughly. The mixture was then diluted to the 100 mL mark with methanol, shaken uniformly, and ultrasonicated in an ultrasonic cleaner (KQ-700DV, Kunshan Ultrasonic Instruments Co., Ltd., Kunshan, China) for 15 min. The solution was filtered through a 0.45 μm organic filter membrane and stored for subsequent use.

Sample preparation: For the hydroponic experiment, 20 mL of soybean nutrient solution was centrifuged at 12,000× *g* for 10 min and the supernatant was filtered through a 0.45 μm aqueous filter membrane, transferred to a clean stoppered test tube, and stored in a refrigerator at 4 °C for use. For the pot experiment, soil samples were sieved through a 40-mesh sieve to remove large particles. 10 g of sieved soil sample (accurate to 0.0001 g) was accurately weighed with an analytical balance (BS124S, Sartorius Scientific Instruments Co., Ltd., Beijing, China) and placed in a stoppered Erlenmeyer flask. 50 mL of mixed solvent of methanol and glacial acetic acid (*v*:*v* = 9.5:0.5) was added, and the mixture was ultrasonicated for 30 min. The sample was washed with methanol and filtered into a centrifuge tube, then centrifuged at 2500× *g* for 20 min until the solution became clear. 1 mL of the supernatant was accurately pipetted into a 10 mL volumetric flask, made up to the mark with methanol, shaken well, and ultrasonicated for 5–10 min to fully dissolve the active component. The solution was filtered through a 0.45 μm organic filter membrane and stored for subsequent use.

Analysis of carbendazim residues: The high-performance liquid chromatography (HPLC) system (Agilent Technologies, Santa Clara, CA, USA) operating conditions were as follows: C18 reversed-phase chromatographic column (4.6 mm × 250 mm, 5 μm); mobile phase: methanol/water (*v*:*v* = 55:45); flow rate: 1.0 mL/min; detection wavelength: 280 nm; injection volume: 10 μL; column temperature: 30 °C; retention time: 7.5 min. The mass fraction (*X*) of carbendazim in the test samples was calculated by the following formula:

$$X=\frac{a_{2}m_{1}p}{a_{1}m_{2}}\times 100$$

where: *a*_1_—Peak area of carbendazim in the standard solution;

*a*_2_—Peak area of carbendazim in the test sample;

*m*_1_—Mass of carbendazim in the standard solution, g;

*m*_2_—Mass of the test sample, g;

*p*—Mass fraction of carbendazim in the standard substance, %.

### 4.5. Statistical Analysis

All experimental data were processed and statistically analyzed using SPSS 27.0 software, and graphs were plotted with Origin 2024 software. Differences were analyzed by Duncan’s multiple range test at *p* < 0.05, and the results were expressed as mean ± standard deviation (SD). Spearman correlation analysis was performed in GraphPad Prism 9 to assess the relationships between AMF colonization rate, soybean biomass, the disease index of soybean root rot, and carbendazim residues in the hydroponic and pot experiments. All analyses were based on five treatment groups with three biological replicates per treatment. The correlation results were further visualized using CNSKnowAll platform.

## 5. Conclusions

In the hydroponic experiment, co-inoculation with *R. intraradices* and MHB increased stem diameter, aboveground dry weight, and underground fresh weight by 48.47%, 75.28%, and 23.59%, respectively, compared with carbendazim alone. Carbendazim residues were reduced by 71.84% in the hydroponic experiment and by 81.25% in the pot experiment under co-inoculation. The root rot disease index in the pot experiment decreased by 52.57% relative to the control. Correlation analysis between the two systems revealed significant associations in soybean biomass, disease index, and carbendazim residues. Co-inoculation of *R. intraradices* and MHB therefore enhances soybean biomass, suppresses root rot, and accelerates carbendazim dissipation. These findings provide a theoretical basis for developing microbial biostimulants for greener soybean production. Future field trials are needed to validate the efficacy of this co-inoculation strategy under real cropping conditions and to explore its environmental adaptability and mechanisms, thereby promoting its industrial application and sustainable development.

## Figures and Tables

**Table 1:** Effects of different treatment groups on carbendazim residues in the hydroponic experiment.

Different Treatments	Initial Level of Carbendazim (mg/L)	Dose of Carbendazim Administered (mg/L)	Carbendazim Residues in Hydroponic Nutrient Solution (mg/L)
HCK	0.00	0.00	0.00 ± 0.00 ^d^
HD	0.00	50.00	49.76 ± 0.15 ^a^
HMD	0.00	50.00	17.84 ± 0.38 ^b^
HRD	0.00	50.00	16.35 ± 0.47 ^b^
HRMD	0.00	50.00	14.01 ± 0.06 ^c^

Notes: HCK (blank control); HD (carbendazim spraying); HRD (carbendazim spraying + *R. intraradices* inoculation); HMD (carbendazim spraying + MHB inoculation); HRMD (carbendazim spraying + *R. intraradices* + MHB co-inoculation). Different letters in the same column indicate significant differences at *p* < 0.05 level. All values are expressed as mean ± standard deviation (*n* = 3).

**Table 2:** Effects of different treatment groups on carbendazim residues in the pot experiment.

Different Treatments	Initial Level of Carbendazim (mg/kg)	Dose of Carbendazim Administered (mg/kg)	Carbendazim Residues in Rhizosphere Soil (mg/kg)
PCK	0.00	0.00	0.00 ± 0.00 ^b^
PD	0.00	60.00	56.74 ± 8.73 ^a^
PMD	0.00	60.00	22.26 ± 0.33 ^ab^
PRD	0.00	60.00	11.13 ± 0.21 ^b^
PRMD	0.00	60.00	10.64 ± 0.61 ^b^

Notes: PCK (blank control); PD (carbendazim spraying); PRD (carbendazim spraying + *R. intraradices* inoculation); PMD (carbendazim spraying + MHB inoculation); PRMD (carbendazim spraying + *R. intraradices* + MHB co-inoculation). Different letters in the same column indicate significant differences at *p* < 0.05 level. All values are expressed as mean ± standard deviation (*n* = 3).

## Data Availability

The original contributions presented in this study are included in the article.
